# Spatiotemporal patterns of the macrofaunal community structure in the East China Sea, off the coast of Zhejiang, China, and the impact of the Kuroshio Branch Current

**DOI:** 10.1371/journal.pone.0192023

**Published:** 2018-01-31

**Authors:** Yong Xu, Fei Yu, Xinzheng Li, Lin Ma, Dong Dong, Qi Kou, Jixing Sui, Zhibin Gan, Lin Gong, Mei Yang, Yueyun Wang, Yue Sun, Jinbao Wang, Hongfa Wang

**Affiliations:** 1 Institute of Oceanology, Chinese Academy of Sciences, Qingdao, China; 2 University of Chinese Academy of Sciences, Beijing, China; 3 Laboratory for Marine Biology and Biotechnology, Qingdao National Laboratory for Marine Science and Technology, Qingdao, China; 4 Laboratory for Ocean and Climate Dynamics, Qingdao National Laboratory for Marine Science and Technology, Qingdao, China; 5 Second Institute of Oceanography, State Oceanic Administration, Hangzhou, China; National Taiwan Ocean University, TAIWAN

## Abstract

The Kuroshio Current intrudes in the bottom layer of the East China Sea continental shelf from the northeast of Taiwan via two bottom branches named the Nearshore Kuroshio Branch Current (NKBC, along the 60 m isobath) and the Offshore Kuroshio Branch Current (OKBC, along the 100 m isobath). However, knowledge on the macrofaunal responses to these bottom branches is limited. This study examined the variations in the benthic macrofaunal community in a section of the East China Sea under the influence of the NKBC. Seven sites corresponding to three regions (the west, middle and east region) were sampled using an Agassiz trawl net at a monthly rate from February to November 2015 (except in August). A total of 270 macrofaunal species were collected in this study. Cluster analysis and nMDS ordination revealed three communities: the inshore, Kuroshio and offshore communities, roughly corresponding to the west, middle and east of NKBC route. Significant differences in the species composition (one-way PERMANOVA) and diversity indices (one-way ANOVA) among the regions and communities were observed, while no statistically significant difference among the months was detected. The indicator species also varied among the communities, with *Sternaspis scutata* and *Odontamblyopus rubicundus* dominating the inshore community, *Camatopsis rubida*, *Schizaster lacunosus* and *Craspidaster hesperus* dominating the Kuroshio community, and *Portunus argentatus*, *Champsodon snyderi* and *Coelorinchus multispinulosus* dominating the offshore community. Some rare species (e.g., *Neobythites sivicola*) may indicate the passage of the NKBC better than the indicator species. A redundancy analysis was used to describe the relationship between the macrofaunal species and environmental variables in this study. Water depth and turbidity played important roles in the distribution of the macrofauna. *S*. *scutata* and *O*. *rubicundus* were associated with high turbidity and shallow depth, while *Plesionika izumiae* and *P*. *argentatus* were associated with low turbidity and deep depth. This study outlines the impact of the NKBC on the distribution patterns of the macrofaunal community of the East China Sea. More studies are needed to understand the detailed interactions between macrofauna and the NKBC in the future.

## Introduction

**Fig 1:**
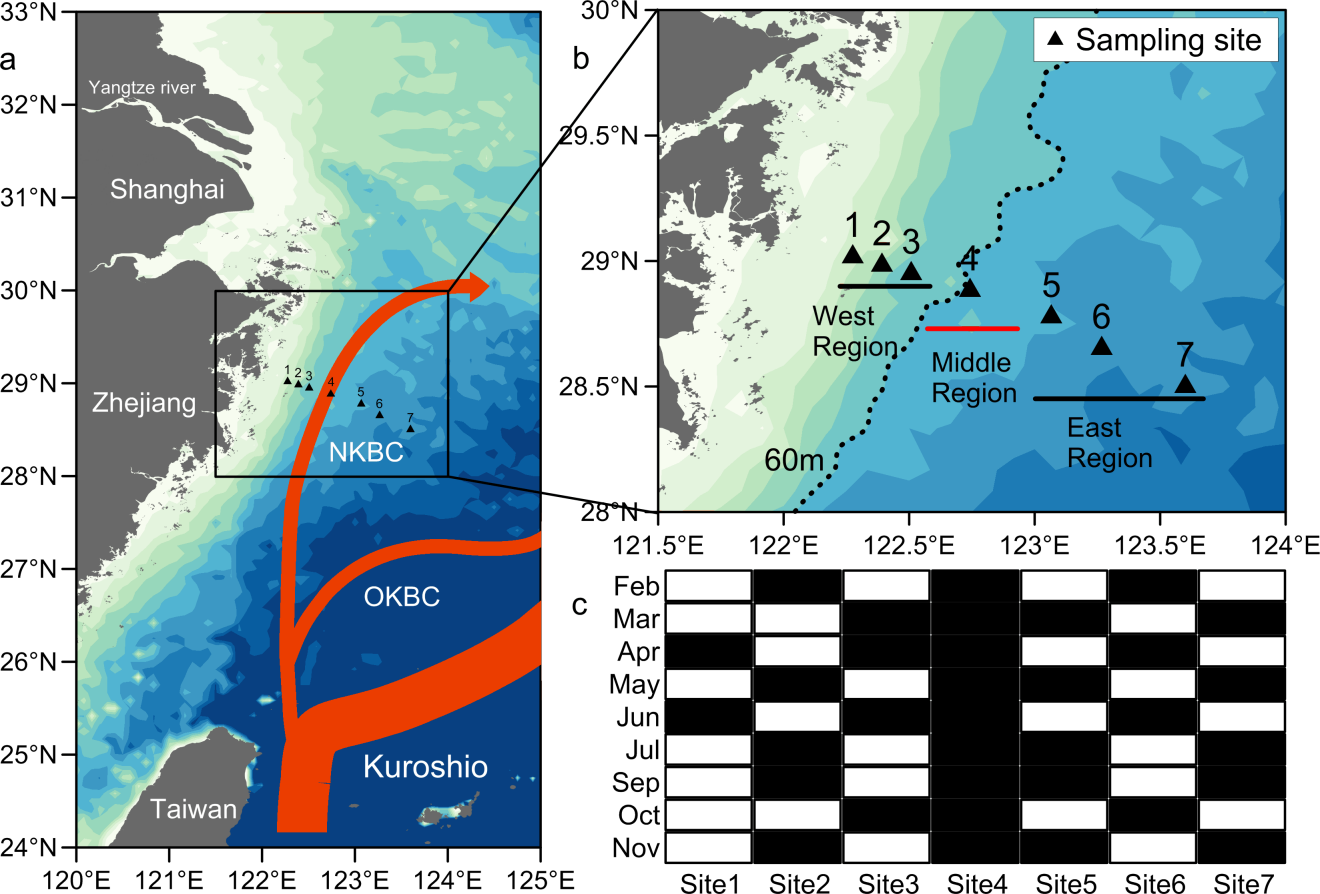
Location map of the sampling sites in the East China Sea. (a) Kuroshio and its branches (NKBC: Nearshore Kuroshio Branch Current; OKBC: Offshore Kuroshio Branch Current), as suggested by Yang et al. [11] and Wang et al. [13]. (b) Seven sampling sites corresponding to three regions (Site 1–3: the west region; Site 4: the middle region; Site 5–7: the east region). (c) Sampling procedure for each month (the black rectangle: physical, chemical and biological sampling site; the white rectangle: only physical and chemical sampling site).

The East China Sea (ECS) has the most extensive continental shelf in the northwestern Pacific Ocean and covers an area of 7.7 × 10 km^2^. The hydrological characteristics in the ECS shelf are very complex because of the influence of the coastal water, the Yangtze River-diluted water and the Kuroshio Current. Physical and chemical characteristics, such as depth, salinity, nutrients and chlorophyll a content, are related to water masses [1–2]. Marine plankton, such as nanoflagellates [3], planktonic ciliates [4–5] and copepods [6–9], also exhibit different patterns under different water masses. However, most of the water mass-biota relationships in the ESC shelf that were identified by previous studies were influenced by the surface water and plankton species. Recently, Yang et al. (2011) found the Kuroshio Current intruding across ECS shelf bottom water via the Kuroshio Bottom Branch Current to the northeast of Taiwan (KBBCNT) [10]. The intrusion pattern of the Kuroshio Current includes the Nearshore Kuroshio Branch Current (NKBC, along the 60 m isobath off the coast of Zhejiang province, Fig 1A) and the Offshore Kuroshio Branch Current (OKBC, along the 100 m isobath) [11]. The NKBC forms the bottom saline water off the coast of Zhejiang province, China [10]. High concentrations of nutrients originating from the Kuroshio Current were also detected off the coast of Zhejiang based on numerical experiments and observations [12]. Wang et al. (2016) further confirmed the existence of the NKBC and OKBC by analysing the nitrate isotopic composition of the bottom water of the ECS shelf [13]. However, no biological responses to the NKBC have been studied, although the recognition of these responses is very important. In this study, we hypothesized that the NKBC had a significant impact on the benthic macrofaunal community and analysed the responses of the macrofauna to the NKBC.

Benthic macrofauna represent an important component of marine systems and play crucial roles in biological and chemical processes. They can promote the decomposition of organic matter and accelerate the transformation and circulation of nutrients in food webs [14]. Some macrofauna are indicators of the deterioration of the marine environment [15]. Macrofaunal compositions, like the composition of plankton, can also reflect the hydrological regime, although benthic macrofauna are relatively stable compared to plankton and nekton. For example, in the adjacent Yellow Sea, the macrofaunal composition in the Yellow Sea Cold Water Mass was different from that in other water masses [16–18]. Investigations on the benthic macrofauna in the ECS can be traced back to the late 1950s. Liu et al. [16] first illustrated the benthic macrofaunal community in the ECS shelf based on samples mainly collected in the late 1950s (grab and Agassiz trawl data) and noted that the macrofaunal composition and distribution patterns reflected the characteristics of the marine environment in the ECS. After that, many surveys were carried out on the macrofauna [19–20], but few studies analysed the relationship between the macrofauna and water masses in the ECS because of a lack of the knowledge on the hydrological regime at the bottom of the ECS shelf. Only after the existence of the Kuroshio bottom branch currents (NKBC and OKBC) was suggested did researchers start to analyse their influence on marine benthic organisms. For example, Xu et al. [21] found that the macrofaunal community showed a depth gradient off the coast of Zhejiang province using Agassiz trawl data and revealed that some species may reflect the influence of the Kuroshio Current. However, more evidence is needed to confirm the influence of the NKBC on the macrofauna as no study directly links the NKBC and the macrofauna.

The present study aims to (1) analyse the spatial and temporal patterns of the macrofaunal community and diversity in and around the NKBC in the ECS; (2) identify indicator species; and (3) detect the environmental variables significantly influencing the macrofaunal community.

## Materials and methods

### Study area and sampling design

To study the spatial and temporal variations in the macrofaunal community and the influence of the NKBC, a section comprising seven sites (site 1: 122°16′30″E, 29°01′00.12″N; site 2: 122°23′30.12″E, 28°58′59.88″N; site 3: 122°30′29.88″E, 28°57′00″N; site 4: 122°44′30.12″E, 28°52′59.88″N; site 5: 123°04′00.12″E, 28°46′39″N; site 6: 123°16′00.12″E, 28°39′06.12″N; site 7: 123°36′00″E, 28°30′00″N) was studied in the ECS, off the coast of Zhejiang in China (Fig 1). Site 4 was situated along the 60 m isobath, where the NKBC passes through. Sites 1–3 were shallower and sites 4–7 were deeper than site 4. These sampling sites corresponded to three regions, which were labeled the west (sites 1–3), the middle (site 4) and the east (site 5–7) regions (Fig 1B). Seven sites were sampled once a month for physical and chemical variables during February to November 2015, except during August. For the biological variables, three or four sites (at least one site in each region) were sampled once per month (Fig 1C).

The macrofauna were collected using a 1.5 m × 0.5 m Agassiz trawl net. The mesh size decreased from the net body to the cod end, with the largest size, 20 mm, at the mouth of the net and the smallest size, 7 mm, at the cod end. The sled was towed along the sea bottom for approximately 15 min with a velocity of 2–3 knots (approximately 4–6 km/h). At the beginning and the end of each tow, the geographical position was obtained using GPS. The biotic samples were preserved in 75% alcohol on board after collection. In the laboratory, the samples were identified to the lowest possible taxonomic level, counted and weighed. A 0.001 g precision electric balance was used to obtain the wet weights (shells included for Mollusca). Physical and chemical variables, including the temperature (°C), salinity, conductivity (S/m), density (kg/m^3^), dissolved oxygen (mg/L), turbidity (NTU) and fluorescence (mg/m^3^) were measured *in situ* by probes mounted on a CTD (Sea-Bird SBE911 plus, Sea-Bird Electronics, Inc., Bellevue, Washington, USA) with an interval of 1–2 m above the sea bottom. Among the probes, an SBE43 sensor was used to measure the dissolved oxygen concentration, and a Chelsea fluorometer was used for the fluorescence measurements. The water depth was measured on board with the acoustic reflection technique.

### Ethics statement

The investigations involving the collection of macrofauna were approved by the Institute of Oceanology, Chinese Academy of Sciences (IOCAS). In this study, all investigations and visual inspections performed on the macrofauna compiled with the regulations on the use and care of laboratory animals of China. We confirm that no endangered or protected species were involved in this study, and all investigations were performed with the minimum amount of suffering of the macrofauna. All sampling procedures were approved as part of obtaining the field permit by the scientific programme “Western Pacific Ocean System: Structure, Dynamics and Consequences, WPOS”.

### Data analyses

Principal component analysis (PCA) was used to portray the spatial and temporal patterns of the main environmental characters of the studied sites and to explore the relationship among the environmental variables. Sites that were separated by a large distance on the PCA plot had different environmental characteristics. The original environmental data were log_e_(x+1) transformed, centered, and normalized to reduce the data skewness.

The macrofaunal community pattern was examined by cluster analysis and non-metric multidimensional scaling (nMDS) ordination. In this study, only the species with a frequency of occurrence > 5% and an abundance > 0.01 ind./m^2^ were included to minimize the effects of rare species [22–24]. The Bray-Curtis distance matrix (Q mode, for sites) and the Chi-square distance matrix (R mode, for species) were constructed based on square root transformed abundance data. The Ward linkage method was employed in a cluster analysis to define the macrofaunal communities [25]. The cluster analysis was combined with a heatmap to visualize the occurrence pattern of the macrofauna, which was suitable as an initial exploratory tool for the collected data [24]. To examine the significant differences in the species composition of the communities, regions and months, a permutational multivariate analysis of variance (PERMANOVA) test [26] and multiple comparisons with the Bonferroni correction were performed.

The number of species (*S*), Margalef richness index (*d*), Shannon-Wiener index (*H*’, log_2_), Pielou’s evenness index (*J*’), abundance (×10^3^ ind./km^2^) and biomass (kg/km^2^) values were calculated and their difference significance among the communities, regions and months were detected using one-way ANOVA. A post hoc comparison was performed with the Tukey HSD method if any significance was found during the ANOVA. Before the analyses, these univariate biotic variables were tested for normality and homogeneity of variance using the Shapiro-Wilk test and Bartlett test, respectively. To determine the normality and homogeneity of variance, *S* was log_e_(x+1) transformed for the community comparison, and abundance and biomass values were log_e_(x+1) transformed for both the community and regional comparisons. For the month comparison, the abundance was tested using the Kruskal-Wallis rank sum test because of the non-normality of the data, even after transformation.

The indicator species in each community were identified using the Indicator Value Index (IndVal) [27]. This index was obtained with the formulas below:

SP_*ji*_ = Nindividuals_*ji*_/Nindividuals_*i*_
FI_*ji*_ = Nsites_*ji*_/Nsites_*i*_
IndVal_*ji*_ = SP_*ji*_ × FI_*ji*_ × 100

where SP_*ji*_ is the specificity of species *i* to community *j*. Nindividuals_*ji*_ is the mean abundance of species *i* in community *j*, and Nindividuals_*i*_ is the sum of the mean abundance of species *i* among all communities. FI_*ji*_ is the fidelity of species *i* to community *j*. Nsites_*ji*_ is the number of sites in community *j* where species *i* occurs, and Nsites_*i*_ is the total number of sites in community *j*.

The significance of the IndVals for each community was examined using the Monte Carlo randomization test (999 permutations).

To identify the environmental variables that most influenced the macrofaunal community, a redundancy analysis (RDA) model was constructed. The Hellinger transformation was performed for the species abundance matrix (only species with a frequency of occurrence > 5% and an abundance > 0.01 ind./m^2^ were included) and a log_e_(x+1) transformation was performed for the environmental matrix before analysis. Significant environmental variables were examined using a forward stepwise selection based on the Monte Carlo permutation test (999 permutations) and Akaike information criteria (AIC).

All statistical analyses were performed in the R computing environment (R Development Core Team, 2011) with R packages “ade4” [28], “vegan” [29], “pheatmap” [30], “labdsv” [31], and “ggplot2” [32].

## Results

### Environmental variables

**Fig 2:**
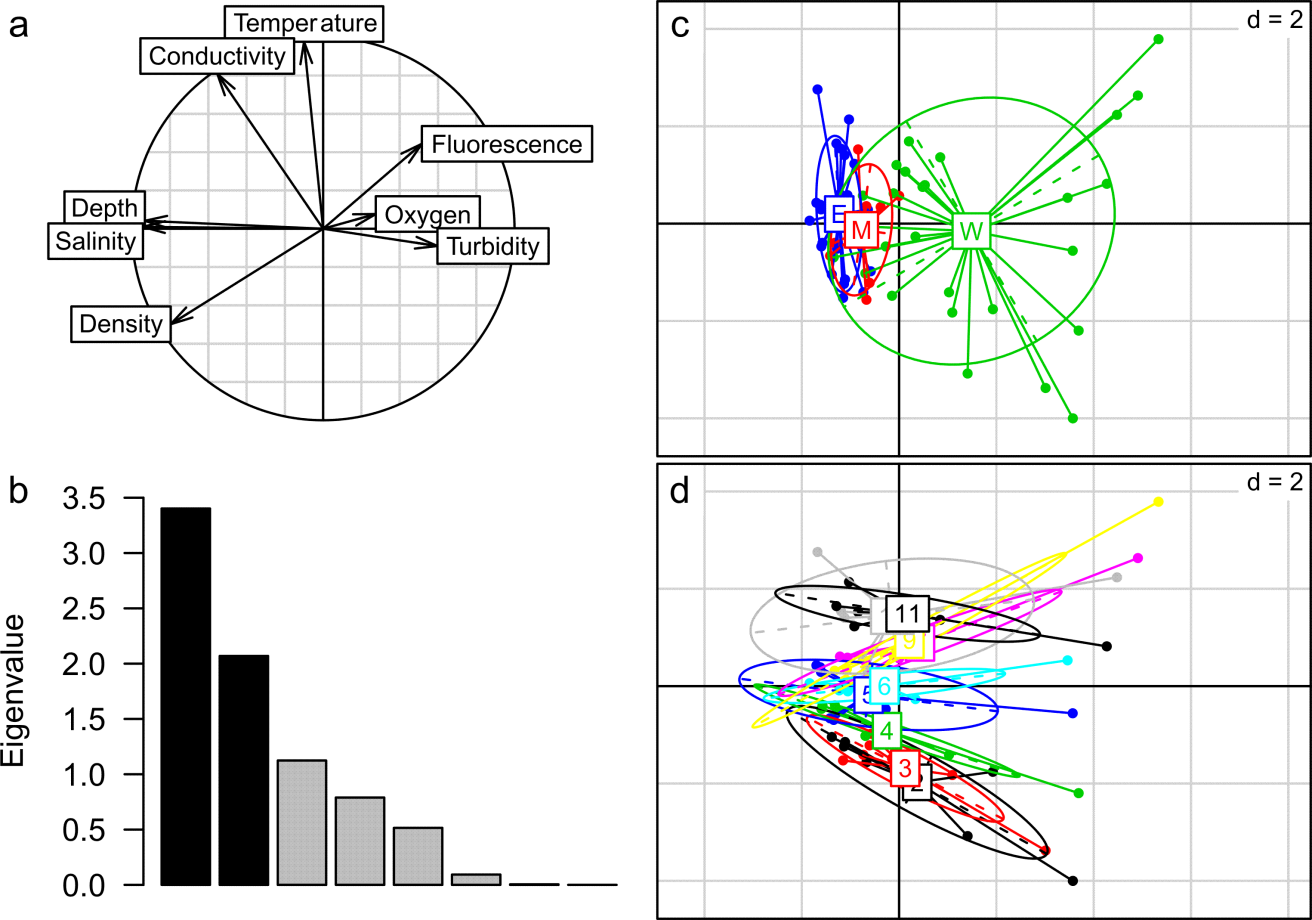
Principal component analysis (PCA) plots for the environmental variables. Correlations of the environmental variables (a), eigenvalues (b), and multivariate analyses of the environmental variables through a scatter diagram of regions (c) and months (d).

Changes in the environmental variables are shown in Fig 2. Depth, salinity and density were highly correlated with each other but negatively associated with the fluorescence and turbidity. These variables were linked to axis 1, explaining 42.53% of the total variance. Temperature and conductivity were linked to axis 2, accounting for 25.87% of the total variance. Most environmental variables exhibited significant regional gradients along axis 1, with the east region and the middle region characterized by high water depth and salinity, whereas the west region featured high fluorescence and turbidity (Fig 2C). Significant monthly gradients along axis 2 were also observed, with February, March and April characterized by low temperature and other months (except May and June) characterized by high temperature (Fig 2D).

### Macrofaunal community structure

**Fig 3:**
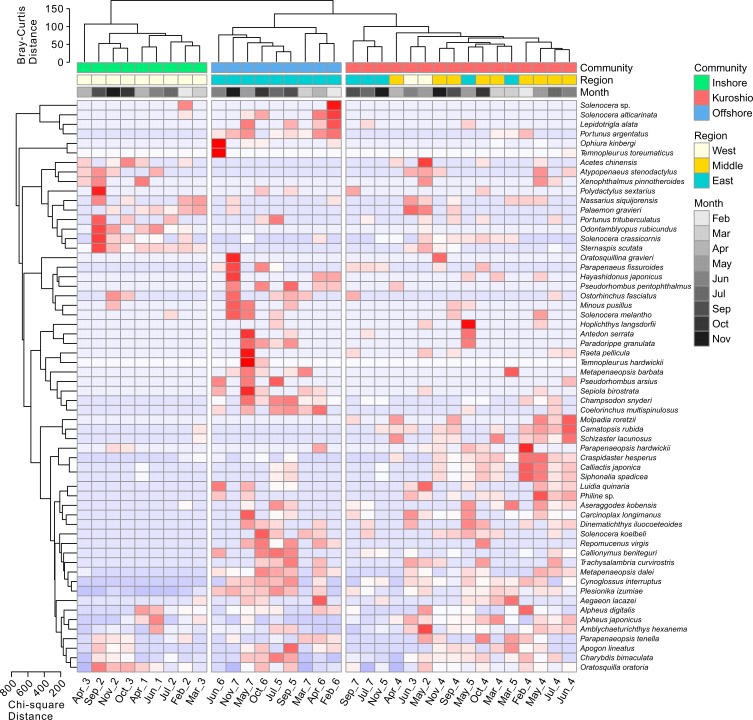
The distribution of macrofauna in the sampling sites. The dendrogram shows the similarity relationship of the sites (Bray-Curtis distance, Q mode) and species (Chi-square distance, R mode) based on Ward’s hierarchical clustering method. The heatmap shows the square root-transformed abundance data standardized by rows. The white and blue colours indicate weak correlations (low ratios) between the species abundances and sampling sites, while the pink and red colours show strong correlations (high ratios).

**Fig 4:**
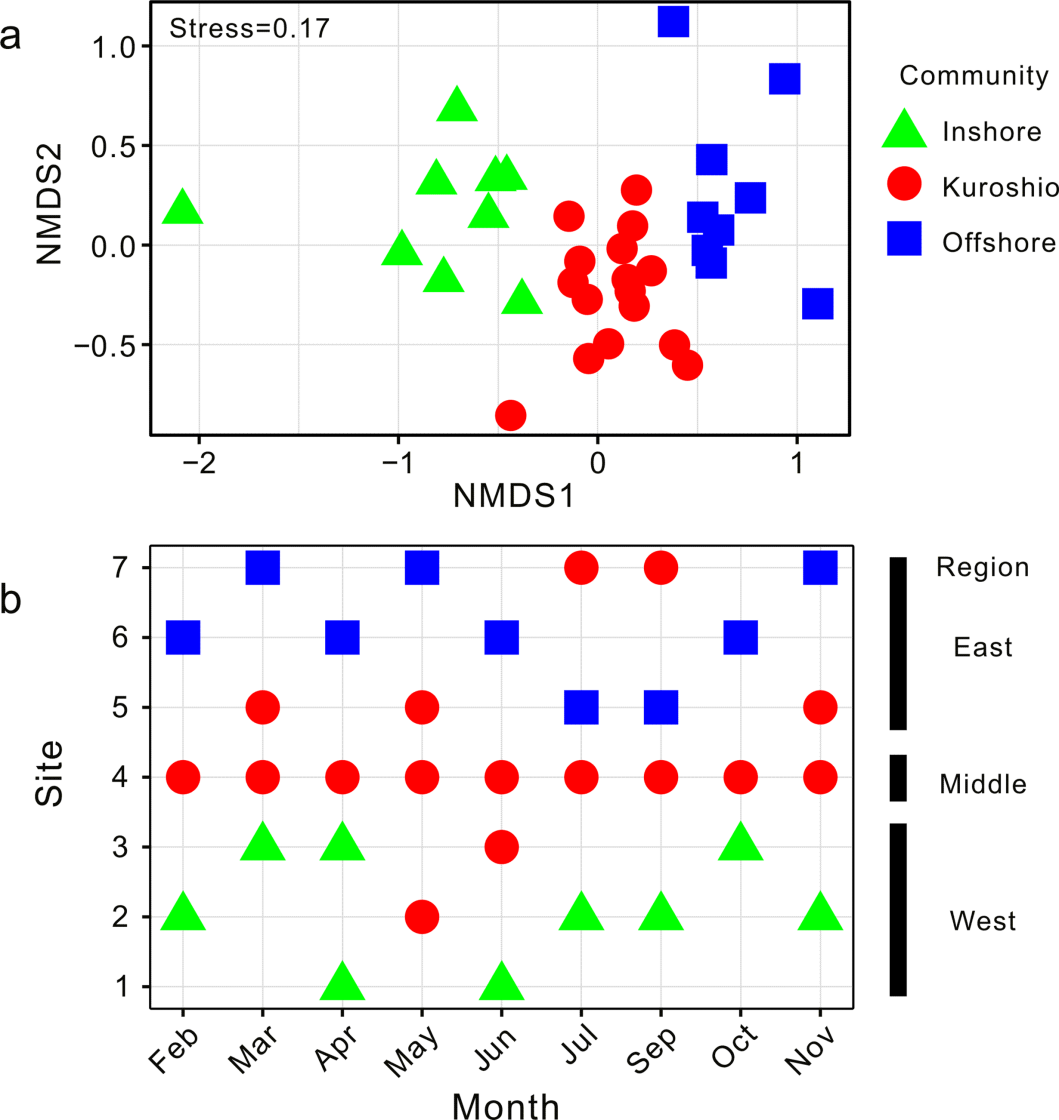
Non-metric multidimensional scaling ordinations (nMDS) for macrofauna (a) and the spatial distribution of each community (b) in the study area.

Ward’s hierarchical cluster analysis based on the Bray-Curtis distance suggested that three communities existed in the study area, the inshore community, Kuroshio community (because of the passing of the NKBC) and offshore community (Fig 3). They were located roughly from the inshore to the offshore regions. The inshore community was composed of the samples collected in the west region, the offshore community was composed of the samples collected in the east region, and the Kuroshio community was mainly composed of the samples collected in the middle region (Figs 3 and 4B). The two-dimensional nMDS ordinations also confirmed the identification of the three communities, with evident distinctions along the first axis (Fig 4A).

One-way PERMANOVA revealed significant differences in the species composition among the communities (*F*_2, 33_ = 6.406, *P* < 0.001), and pairwise comparisons showed significant variations between all pairwise combinations of the three communities (inshore-Kuroshio: *P*_adjusted_ = 0.003; inshore-offshore: *P*_adjusted_ = 0.003; Kuroshio-offshore: *P*_adjusted_ = 0.003). Significant regional variations in species composition were also detected by one-way PERMANOVA and subsequent pairwise comparisons (*F*_2, 33_ = 5.677, *P* < 0.001; west-middle: *P*_adjusted_ = 0.003; west-east: *P*_adjusted_ = 0.003; middle-east: *P*_adjusted_ = 0.003). However, no significant monthly differences in the species composition were observed by one-way PERMANOVA (*F*_8, 33_ = 1.241, *P* = 0.080).

### Community analyses at spatial and temporal scale

**Fig 5:**
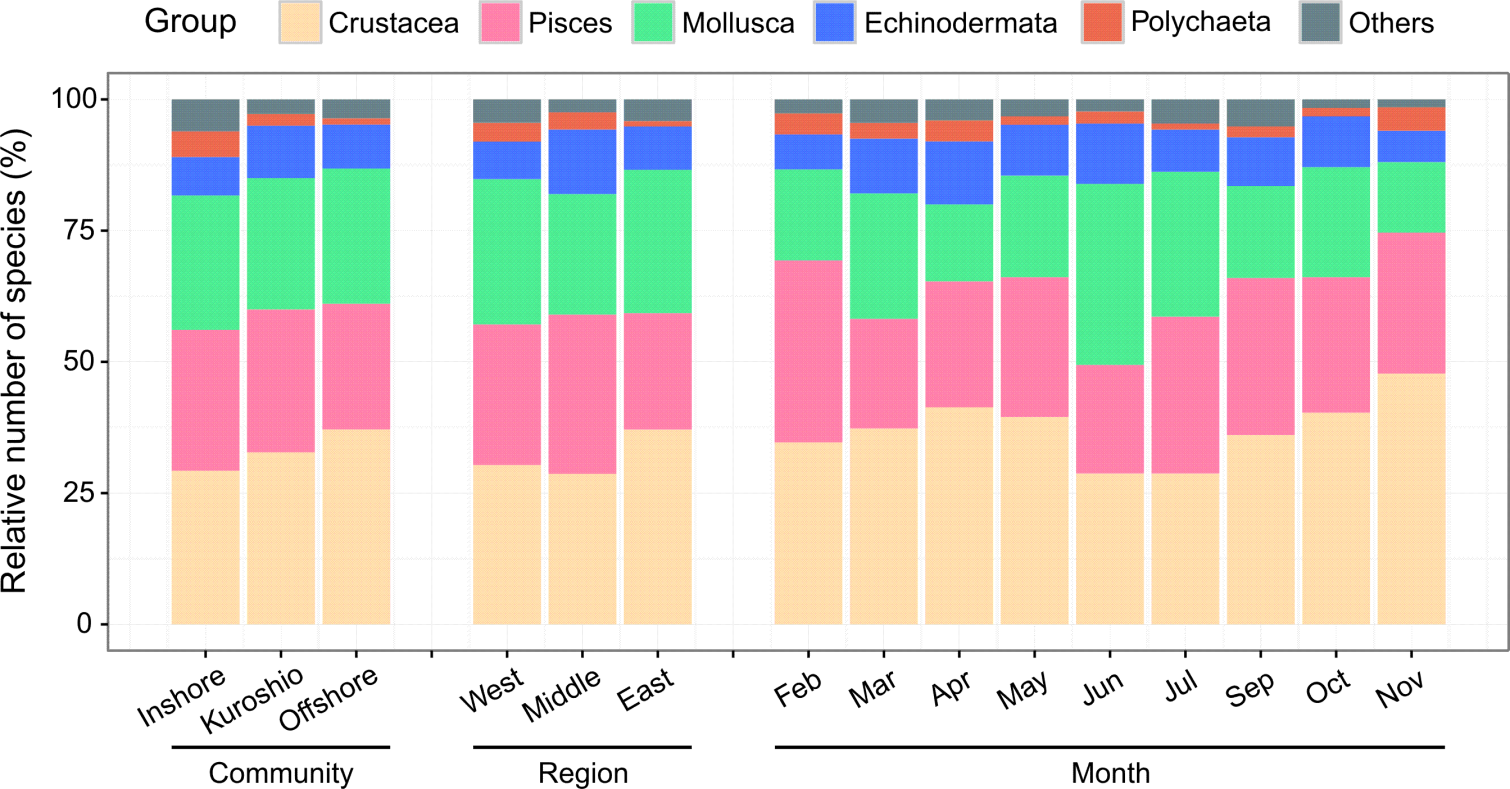
Relative number of species of major taxonomic groups for communities, regions and months.

In total, 270 species of macrofauna were identified from the study area during all nine cruises. Crustacea (32.96% of the total species, 89 species) and Mollusca (27.78%, 75 species) dominated the macrofauna, followed by Pisces (23.33%, 63 species), Echinodermata (10%, 27 species), Polychaeta (2.59%, 7 species) and others (Nemertinea and Cnidaria, 3.33%, 9 species). The relative number of species of the major taxonomic groups for the communities, regions and months are shown in Fig 5, with Crustacea, Piseces and Mollusca being the predominant groups (accounting for 80% or more of all macrofauna). For the communities, the relative number of Crustacea species was highest in the offshore community and lowest in the inshore community, while the relative number of Polychaeta species was highest in the inshore community and lowest in the offshore community. In the middle region, the relative numbers of Pisces and Echinodermata species were higher than those of the other two regions, while the relative numbers of Mollusca and Crustacea species were lower. The monthly variation showed that the relative number of Crustacea species was the lowest in the summer months (June and July), while the opposite was true for Mollusca.

The diversity indices, abundance and biomass values are shown in Table 1. Significant differences among the communities were detected for the number of species (*S*), Margalef richness index (*d*), abundance and biomass using one-way ANOVA (*S*: *F*_2, 33_ = 14.960, *P* < 0.001; *d*: *F*_2, 33_ = 15.100, *P* < 0.001; abundance: *F*_2, 33_ = 4.756, *P* = 0.016; biomass: *F*_2, 33_ = 4.702, *P* = 0.017). Multiple post hoc comparisons showed that *S*, *d* and biomass in the Kuroshio and offshore communities were significantly higher than those of the inshore community (Tukey HSD, *P* < 0.05), and the abundance in the offshore community was significantly higher than that in the inshore community (Tukey HSD, *P* < 0.05). Significant differences among the regions were also observed for *S* and *d* (*S*: *F*_2, 33_ = 5.757, *P* = 0.007; *d*: *F*_2, 33_ = 8.017, *P* = 0.002). However, for all biotic parameters, no differences were detected among the months (*P* > 0.05).

**Table 1:** Number of species (*S*), Margalef richness index (*d*), Shannon-Wiener index (*H*’, log_2_), Pielou’s evenness index (*J*’), abundance (×10^3^ ind./km^2^) and biomass (kg/km^2^) for the communities, regions and months (mean ± SE).

	*S*	*d*	*H'*(log_2_)	*J'*	Abundance	Biomass
**Community**						
Inshore	**18.89±1.84** ^**A**^	**3.75±0.33** ^**A**^	3.18±0.19	0.77±0.04	**123.51±40.95** ^**A**^	**239.77±100.24** ^**A**^
Kuroshio	**33.07±2.26** ^**B**^	**5.64±0.24** ^**B**^	3.43±0.14	0.69±0.03	**225.07±45.28** ^**AB**^	**449.89±67.59** ^**B**^
Offshore	**42.40±4.36** ^**B**^	**6.79±0.53** ^**B**^	3.40±0.30	0.64±0.06	**462.98±162.45** ^**B**^	**611.41±113.22** ^**B**^
**Region**						
West	**22.45±2.99** ^**A**^	**4.12±0.37** ^**A**^	3.15±0.17	0.73±0.04	187.69±62.33	293.90±92.85
Middle	**35.11±2.76** ^**AB**^	**5.90±0.33** ^**B**^	3.69±0.13	0.73±0.03	225.47±41.43	544.72±82.99
East	**37.64±3.78** ^**B**^	**6.27±0.45** ^**B**^	3.30±0.23	0.65±0.04	358.83±123.33	491.78±96.00
**Month**						
Feb	31.67±4.26	5.67±0.21	3.92±0.14	0.79±0.05	187.14±94.16	369.56±161.97
Mar	26.50±3.10	5.03±0.24	3.47±0.22	0.74±0.07	138.02±53.19	181.69±71.69
Apr	22.25±6.87	4.46±0.91	3.24±0.17	0.78±0.05	100.28±48.53	174.78±80.38
May	51.25±5.02	7.81±0.85	3.83±0.44	0.67±0.07	452.96±99.31	780.49±84.92
Jun	31.75±5.22	4.91±0.62	2.65±0.56	0.54±0.11	660.19±396.22	443.52±38.90
Jul	34.25±9.59	5.56±1.08	3.07±0.18	0.64±0.06	226.77±130.52	401.87±219.59
Sep	34.00±6.45	5.61±1.01	3.32±0.37	0.66±0.05	256.95±86.04	670.64±240.12
Oct	30.33±7.84	5.25±1.09	3.60±0.21	0.75±0.03	216.67±80.87	609.82±225.61
Nov	26.00±2.92	5.03±0.53	3.31±0.35	0.71±0.07	141.33±58.27	367.56±116.28

Different uppercase letters (A and B) indicate significant differences

### Indicator value of species for each community

**Fig 6:**
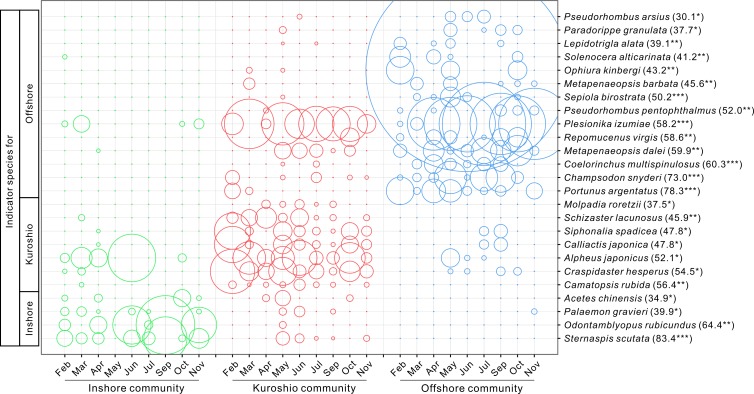
Monthly variations of the abundance of species with significant IndVal for each community. The abundance increases linearly with the area of a circle and the largest circle corresponded to 1.525 × 10^6^ ind./km^2^. The number in the bracket was the IndVal index. *significant at 0.05 level; **significant at 0.01 level; ***significant at 0.001 level.

According to the IndVal index, four indicator species with significant indicator values (IndVal > 25 and *P* < 0.05) were identified for the inshore community and the first three representative species were *Sternaspis scutata*, *Odontamblyopus rubicundus* and *Palaemon gravieri*. Seven indicator species were found for the Kuroshio community, and the first three representative species were *Camatopsis rubida*, *Schizaster lacunosus* and *Craspidaster hesperus*. Fourteen indicator species were detected for the offshore community, and the first three species were *Portunus argentatus*, *Champsodon snyderi* and *Coelorinchus multispinulosus*. The monthly variations in the abundances of the indicator species are shown in Fig 6. *Molpadia roretzii* occurred exclusively in the Kuroshio community while *Solenocera alticarinata* did not occur in the Kuroshio community. *Siphonalia spadicea* and *Calliactis japonica* showed the same variation trends in abundance.

### Relationship between macrofaunal community and environmental variables

**Fig 7:**
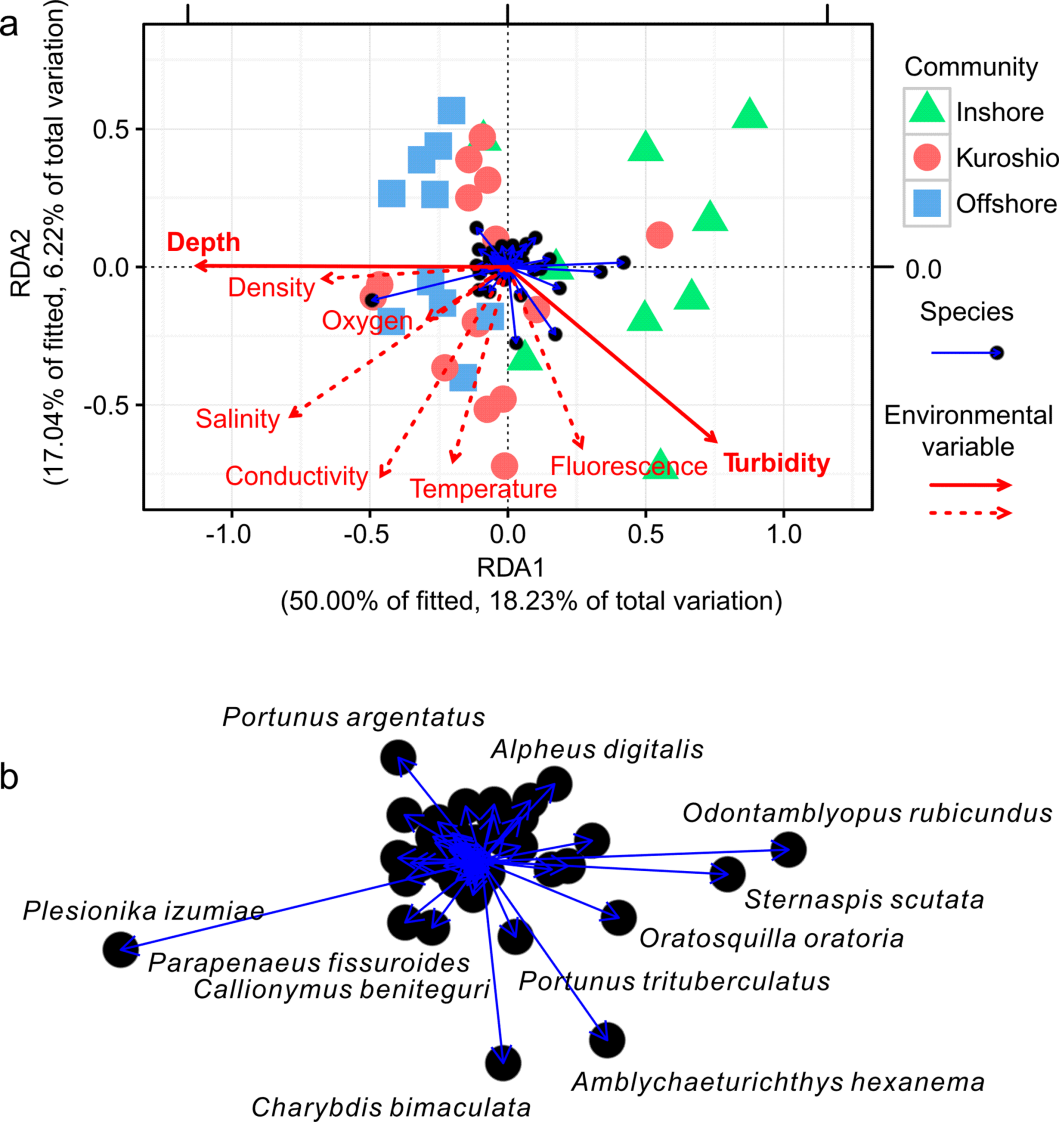
RDA triplot showing relationships between the species and environmental variables (scaling = 2). Solid red lines depict significant environmental variables, while dashed red lines do not in (a). The distribution of species in the RDA triplot is shown in (b).

The relationship between the macrofaunal community and environmental variables was revealed in the RDA triplot (Fig 7). In the RDA model, the unadjusted and adjusted R^2^ values were 0.365 and 0.161, respectively. The Monte Carlo permutation tests (999 permutations) showed that the RDA model including all environmental variables was very significant (*Pseudo-F* = 1.794, *P* = 0.001). The first canonical axis explained 50% of the total variance of the data, and the second axis accounted for 17% (Table 2). The marginal tests of the axes (999 permutations) revealed that the *P* values of the first two axes were relatively low and could indicate a good separation along the axis for the samples and species. The details of the RDA model are shown in Table 2.

**Table 2:** Summary of the RDA analysis.

	RDA1	RDA2	RDA3	RDA4	RDA5	RDA6
*F*	7.1757	2.4458	1.6817	1.0104	0.7018	0.5452
*P* value	0.001***	0.005**	0.061	0.428	0.747	0.930
Eigenvalue	0.1019	0.0347	0.0239	0.0144	0.0100	0.0077
Proportion explained	0.5000	0.1704	0.1172	0.0704	0.0489	0.0380
Cumulative Proportion	0.5000	0.6704	0.7875	0.8579	0.9068	0.9448
Depth	-0.9758	0.0035	0.0376	-0.1300	-0.1629	0.0139
Temperature	-0.1721	-0.6048	-0.3281	0.4342	-0.1501	-0.1621
Conductivity	-0.3980	-0.6507	-0.2622	0.2620	-0.2893	-0.1873
Salinity	-0.6809	-0.4653	0.0691	-0.1193	-0.4502	-0.1182
Density	-0.5793	-0.0367	0.3750	-0.3833	-0.3545	0.0131
Oxygen	-0.2510	-0.1647	-0.0603	-0.5337	0.6736	0.3053
Fluorescence	0.2307	-0.5610	-0.0904	0.1920	-0.0968	0.6352
Turbidity	0.6509	-0.5435	0.1341	0.3305	0.1370	-0.2664

** = *P* < 0.01

*** = *P* < 0.001.

The RDA model revealed that the depth and turbidity were significant environmental variables (Monte Carlo permutation tests with 999 permutations, *P* < 0.05) influencing the macrofaunal community and were closely correlated with RDA1 (Fig 7A, Table 2). An obvious gradient for the communities (especially for the inshore community and the offshore community) was divided by RDA1 from the left to right (Fig 7A). The inshore community was characterized by low depth and high turbidity, while the offshore community had high depth and low turbidity. Temperature, fluorescence and conductivity were correlated with RDA2, though the correlation was not significant (*P* > 0.05) based on the Monte Carlo permutation tests (999 permutations). The abundance of *S*. *scutata*, *O*. *rubicundus* and *Oratosquilla oratoria* were high in the inshore community, in accordance with its high turbidity and low depth. *Plesionika izumiae* and *P*. *argentatus* were associated with low turbidity and high depth and were abundant in the offshore community. *Charybdis bimaculata* and *Amblychaeturichthys hexanema* corresponded to high temperature and turbidity. Other species were located near the original point, showing an appreciation to moderate explanatory variables.

In the RDA model constrained by depth and turbidity, the explanatory variables were closely correlated with RDA1, which explained 80% of the total variance of the data. The unadjusted and adjusted R^2^ were 0.218 and 0.167, respectively. It can therefore be concluded that depth and turbidity explain most of the variations in the data.

## Discussion

Since the discovery of the NKBC, there has been few studies on the influence of NKBC on the distribution of marine organisms, although the understanding of this subject is essential for the management of the coastal sea area of the ECS. In this study, we conducted monthly investigations of benthic macrofauna using an Agassiz trawl net at the 60 m isobath off the Zhejiang coast, through which the NKBC passes, and at the side regions. Most of the species collected by the Agassiz trawl net were epibenthos, which have a relatively high motility that enables them to react to hydrographical changes.

### Macrofaunal community and the influence of NKBC`

The macrofaunal composition in the regions that were investigated showed an obvious spatial variation from the west to the east with a depth gradient. The diversity indices (*S* and *d*) also showed significant spatial differences (Table 1). Other studies have also observed the distribution pattern of the ECS macrofauna and reported that the environmental characteristics of the ECS showed obvious depth gradients [16,21,33]. In this study, the environmental variables measured in the middle and east regions were not as distinctive as the species composition (Figs 2C and 3) because of the similar significant *P*_adjusted_ values reported between each region pair during the post hoc comparisons for the species composition following PERMANOVA. This indicates that the species in the middle region have some relation with the NKBC, as this bottom current carries nutrients, such as phosphate, that could enhance productivity and thus provide more food for the species in the region [12,34]. In addition, it may directly bring Kuroshio-oriented macrofauna, contributing to the difference the species composition of the middle and east regions. In this study, three communities (the inshore community, Kuroshio community and offshore community) were identified roughly from the inshore to offshore part of the ECS by cluster analysis and nMDS ordination (Figs 3 and 4), with diversity indices (*S* and *d*) showing significant differences among them (Table 1). The spatial patterns of the three communities varied with time (Fig 4B). For example, the Kuroshio community contained an inshore site (shallower than 60 m) in May and June and the most offshore site in July and September. These monthly patterns of the Kuroshio community coincided with the seasonal variations of the NKBC, as suggested by physical oceanography researchers who indicated that the intrusion of the NKBC strengthened during the late spring, was strongest in the summer, weakened in the fall and became the weakest in the winter [35]. Thus, the hypothesis proposed in the introduction of this work, that the NKBC had a significant impact on the benthic macrofaunal community, can be accepted. For the whole region, the species composition was relatively stable, as no significant monthly difference was detected by PERMANOVA, and no diversity indices showed any significant differences among the months (Table 1). The Kuroshio community existed in each month (Fig 4B), so we may speculate that the NKBC was present throughout the year.

### Indicator species

In this study, the indicator species for the three communities were identified using the IndVal index. The abundance of these species differed greatly among communities (Fig 6). The indicator species *Odontamblyopus rubicundus* mainly occurred in the inshore community. This is a coastal fish species with a low trophic and spatial niche width, feeding mainly on gammarids, bivalves and polychaetes [36–37]. It occurred only once in the Kuroshio community at site 2 in May when the NKBC strengthened [35] and was absent from the NKBC at other times. *Champsodon snyderi* and *Coelorinchus multispinulosus* were typical offshore demersal fish in the ECS shelf [38–39] and were identified as indicator species of the offshore community in this study. Researchers have found that *C*. *snyderi* can be influenced by hydrological conditions, with optimal salinity and depth values of 34.3–35.2 and 70–110 m, respectively [38]. Both fish species were also collected at the coast of Taiwan Island [40], so their presence might reflect, to some extent, the influence of the intrusion of the Kuroshio Branch Current to the ECS shelf. In this study, they could not exactly indicate the route of the NKBC, perhaps because of their limits of depth tolerance, as mentioned above. For the Kuroshio community, the indicator species *Siphonalia spadicea* and *Calliactis japonica* had the same abundances because *C*. *japonica* clung to the shells of *S*. *spadicea*, and there was therefore a one-to-one correspondence between the two species. This phenomenon has not been reported before. There were no indicator species that occurred exclusively in one community except for *Molpadia roretzii*, which was only collected from the Kuroshio community during the spring and summer months, when the NKBC strengthened. However, *M*. *roretzii* is not a Kuroshio-oriented species, as it has been previously found in other coastal areas [41–42]. Indeed, none of the indicator species of the Kuroshio community were Kuroshio oriented, indicating that most macrofauna do not move passively with the flow of the NKBC, at least not as obviously as the plankton in the surface water [3,5]. However, the multivariate analysis was based on a biotic matrix that excluded rare species with a frequency of occurrence lower than 5% and an abundance less than 0.01 ind./m^2^. For these species, the Kuroshio-oriented demersal fish *Neobythites sivicola* occurred at site 4 (July), 5 (March, July and September) and 7 (November), corresponding to the Kuroshio community and offshore community. It has been suggested that the distribution pattern of this species indicates the routes of the Kuroshio bottom branches [21]. In this study, it mostly occurred in regions where the depth ranged between 60 and 70 m (site 4 and 5), indicating the existence of the NKBC.

### Biological-environmental relationships

The RDA model revealed that the water depth and turbidity were significant environmental variables influencing the macrofaunal communities in this study. Water depth has been identified as a key impact factor in many studies investigating submarine macrofaunal distributions, and most macrofauna show a depth gradient in species composition [17,20,43–45]. Previous studies have suggested that other environmental variables, such as temperature, salinity and sediment type, are related to water depth [46]. However, a relationship between the water depth and temperature was not indicated during the PCA and RDA analyses (Figs 2 and 7A). In our study, turbidity showed a negative correlation to water depth (Figs 2 and 7A), perhaps because the huge amount of suspended material carried by the river runoff was diluted with the increase in water depth. It has been shown that high turbidity can have detrimental or negative effects on the diversity and function of benthic organisms [47]. Under experimental conditions, high concentrations of suspended materials incurred mortality in suspension-feeding bivalves, tubicolous polychaetes and deposit-feeding sea urchins; this possibly occurred because the suspended matters interfered with the feeding of suspension feeders by clogging their filter-feeding organs [48]. This may help to explain the low diversity (*S* and *d*) values in the west region and inshore community with high turbidity (Figs 2 and 7A). However, there were still some species (*Sternaspis scutata*, *O*. *rubicundus*, *Charybdis bimaculata* and *Amblychaeturichthys hexanema*) that showed a positive correlation with turbidity. These species were also found to be abundant in the Yangtze River estuary, where large amounts of suspended materials existed [33,49–51]. *C*. *bimaculata* and *A*. *hexanema* have become widespread and predominant in the ECS because of the decline of economic fishes and invertebrates because of overfishing and eutrophication [51–54]. Both species showed a positive but not significant correlation with temperature (Fig 7), and suitable warmer temperatures may accelerate the growth and development of these small, non-economic species. *Plesionika izumiae* and *Portunus argentatus* showed negative correlations with turbidity, perhaps because of the negative effects of turbidity mentioned above. Our results have several implications for the understanding of the impact of the NKBC on the distribution patterns of macrofauna, and more studies will be needed to clarify the mechanisms in the near future.

## Supporting information

S1 Table Species abundance matrix (frequency of occurrence > 5% and abundance > 0.01 ind./m^2^). (XLSX)

S2 Table Environmental matrix. (XLSX)
